# Protein Folding Activity of Ribosomal RNA Is a Selective Target of Two Unrelated Antiprion Drugs

**DOI:** 10.1371/journal.pone.0002174

**Published:** 2008-05-14

**Authors:** Déborah Tribouillard-Tanvier, Suzana Dos Reis, Fabienne Gug, Cécile Voisset, Vincent Béringue, Raimon Sabate, Ema Kikovska, Nicolas Talarek, Stéphane Bach, Chenhui Huang, Nathalie Desban, Sven J. Saupe, Surachai Supattapone, Jean-Yves Thuret, Stéphane Chédin, Didier Vilette, Hervé Galons, Suparna Sanyal, Marc Blondel

**Affiliations:** 1 INSERM U613, Brest, France; 2 Univ Brest, Faculté de Médecine et des Sciences de la Santé, UMR-S613, Brest, France; 3 Etablissement Français du Sang (EFS) Bretagne, Brest, France; 4 CHU Brest, Hop Morvan, Laboratoire de Génétique Moléculaire, Brest, France; 5 CNRS UPS2682, Station Biologique, Protein Phosphorylation & Disease Laboratory, Roscoff, France; 6 Institute of Cell and Molecular Biology, Uppsala University, Uppsala, Sweden; 7 INSERM U648, Laboratoire de Chimie Organique 2, Université Paris Descartes, Paris, France; 8 Institut National de la Recherche Agronomique (INRA), UR892, Virologie Immunologie Moléculaires, Jouy-en-Josas, France; 9 Laboratoire de Génétique Moléculaire des Champignons, IBGC UMR CNRS 5095, Université de Bordeaux 2, Bordeaux, France; 10 Department of Medicine/Biochemistry, University of Fribourg, Fribourg, Switzerland; 11 Department of Medicine, Dartmouth Medical School, Hanover, New Hampshire, United States of America; 12 Department of Biochemistry, Dartmouth Medical School, Hanover, New Hampshire, United States of America; 13 CEA, iBiTec-S, Gif- sur-Yvette, France; Universität Heidelberg, Germany

## Abstract

**Background:**

6-Aminophenanthridine (6AP) and Guanabenz (GA, a drug currently in use for the treatment of hypertension) were isolated as antiprion drugs using a yeast-based assay. These structurally unrelated molecules are also active against mammalian prion in several cell-based assays and *in vivo* in a mouse model for prion-based diseases.

**Methodology/Principal Findings:**

Here we report the identification of cellular targets of these drugs. Using affinity chromatography matrices for both drugs, we demonstrate an RNA-dependent interaction of 6AP and GA with the ribosome. These specific interactions have no effect on the peptidyl transferase activity of the ribosome or on global translation. In contrast, 6AP and GA specifically inhibit the ribosomal RNA-mediated protein folding activity of the ribosome.

**Conclusion/Significance:**

6AP and GA are therefore the first compounds to selectively inhibit the protein folding activity of the ribosome. They thus constitute precious tools to study the yet largely unexplored biological role of this protein folding activity.

## Introduction

Prion-based diseases are fatal neurodegenerative disorders for which no efficient treatment is currently available [1]. These diseases are caused by proteinaceous infectious particles termed prions. According to the “protein only” hypothesis, prions are composed solely of an abnormal form of the PrP protein, a GPI anchored protein normally present at the surface of numerous cell types including neurons. PrP exists in two forms, a “normal” form (PrP^C^) and a pathological, misfolded and protease resistant form (PrP^Sc^). During the course of disease, PrP^Sc^ accumulates and is capable of converting the normal PrP^C^ form to this altered conformation [2]. PrP^Sc^ presents an increase in β-sheet structure [3] and forms aggregates [4]. Prion-based diseases are thus related to other protein misfolding disorders such as Alzheimer, Parkinson or Huntington diseases, which are all characterized by the accumulation of intracellular or extracellular β-sheet rich protein aggregates.

Prions also exist in fungi. In 1994, Reed B. Wickner provided genetic evidence that a long known genetic element of the budding yeast *Saccharomyces cerevisiae* with unusual cytoplasmic inheritance was in fact a prion [5]. Other prions were then discovered in yeast and in filamentous fungi (reviewed in [6]) and several simple reporter systems have been developed to investigate their behavior [7], [8]. These model systems provided direct support for the “protein-only” hypothesis and indicated that fungal prions correspond to self-propagating amyloid or amyloid-like assemblies [9]–[13].

Since prion replication corresponds to the propagation of a misfolded state, the chaperone network plays a central role in prion appearance and maintenance [14]. The chaperone network controls proper folding of newly synthesized proteins, assists assembly of macromolecular complexes and promotes clearance of protein aggregates. The protein chaperone activity is carried out by soluble chaperones and ribosome-associated chaperones. In addition, the ribosome itself was found to possess an intrinsic protein folding activity [15]–[26]. In yeast, prion propagation was demonstrated to be critically dependent on the chaperone machinery, and in particular on Hsp104p chaperone [27]. In addition it is also modulated by different members of the Hsp70p and Hsp40p family and additional co-chaperones [14].

Several approaches towards the development of prion disease therapies are currently being explored. These include identification of pharmacological drugs, immunotherapeutics and PrP^C^ knockdown by RNA interference [1]. Recently a peptide aptamers approach has been developed [28]. Therapeutics can target PrP^C^, PrP^Sc^ or the conversion process. Among the experimental systems used are animal models, cell culture and cell-free conversion assays. Relatively few studies have used systematic screening approaches of large chemical libraries. Recently we have developed a simple, cost-effective, safe and rapid yeast-based method to screen large chemical libraries for antiprion drugs [29], [30]. In a first screen, molecules are isolated on the basis of their *in vivo* activity against the [*PSI* ^+^] yeast prion. The active compounds are then further tested against [URE3], a second yeast prion which is unrelated to [*PSI* ^+^]. Both screens use a colorimetric detection system in which prion-containing yeast cells form white colonies whereas loss of prion leads to formation of red colonies. Remarkably, a vast majority of molecules identified using this yeast-based assay were also active against mammalian prion *ex vivo* in various mammalian cell-based assays ([29]–[31] and D. V. and M. B. unpublished results) and *in vivo* for the few that were tested (Tribouillard-Tanvier *et al.* in press). This finding not only validates our yeast-based assay for high-throughput screening of antiprion molecules, but also establishes yeast as a relevant model system to study mammalian prion-based diseases. Furthermore, some of the antiprion drugs isolated using mammalian cell-based assays, like Quinacrine (QC) or Chlorpromazine (CPZ), were found to be active in the yeast-based assay [29]. Taken together, these results strongly suggest that at least some prion-controlling mechanisms are conserved from yeast to mammals.

For this reason we sought to identify intracellular targets of two of the most active compounds identified using the yeast-based assay: 6-aminophenanthridine (6AP) and Guanabenz (GA, a drug already on the market for the treatment of hypertension (Tribouillard *et al.*, in press)). We have designed affinity chromatography matrices to identify the drugs interactants. We show that the two unrelated antiprion drugs 6AP and GA interact with ribosome in an RNA-dependent manner and specifically inhibit ribosomal protein folding activity, which is carried by the large ribosomal RNA (23S rRNA in *E. coli*, 25S rRNA in *S. cerevisiae* and 28S rRNA in mammals) of the large subunit. 6AP and GA do however not affect protein synthesis neither *in vivo* nor *in vitro*.

## Results

### 6AP and GA and their inactive derivatives lacking antiprion activity

**Figure 1:**
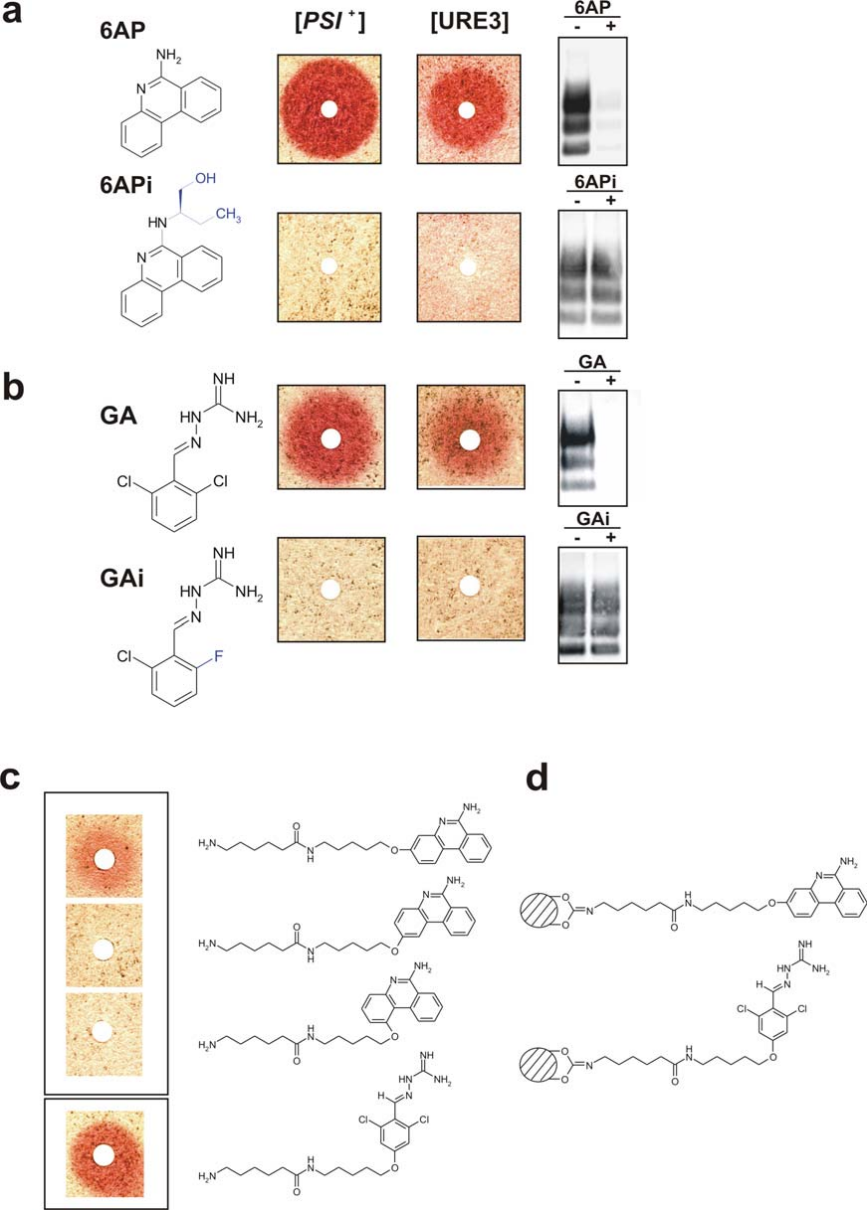
Absence of antiprion activity of 6APi and GAi, two close derivatives of antiprion drugs 6AP and GA and synthesis of affinity matrices for both drugs. a. Left panels: the molecular structure of 6AP and 6APi are depicted. Simple introduction of a 2-(butan-1-ol) group (represented in blue) on the amino group in position 6 is sufficient to abolish the antiprion activity of 6AP. Middle panels: aliquots of an overnight culture of a [*PSI* ^+^] strain (left panel, STRg6) and of a [URE3] strain (right panel, SB34), both growing as white colonies (because they contain prions: see Materials and Methods), were spread on YPD medium supplemented with 200 µM GuHCl. Small filters (similar to the ones used for antibiograms) were placed on the agar surface. 10 µl of a 10 mM solution in DMSO of 6AP (top) and 6APi (bottom) were spotted on the filters. The Petri plates were then incubated three days at 25°C. Red halos were observed around the filters where 6AP was loaded, but not around those where 6APi was spotted indicating that, in contrast to 6AP, 6APi is inactive against yeast prions. Right panels: Scrapie-infected MovS6 cells were treated for six days with DMSO (−) or 20 µM (+) 6AP (upper gel) or 6APi (lower gel) and then lysed. Cell lysates were then subjected to proteinase K digestion (only PrP^Sc^ is resistant to proteinase K) followed by Western blot analysis using an anti-PrP antibody. Note that, as observed with yeast prions, only 6AP was active against PrP^Sc^. b. Same experiments performed with GA and its inactive derivative GAi. Note that only GA is active against both [*PSI*+] and [URE3] yeast prions and mammalian prion in the MovS6 cell-based assay. c. A linker (aminocaproylaminopentyloxy) was attached in position 1, 2 or 3 of 6AP (top 3 structures) or in position 4 of GA (lower structure) as depicted in the molecular structures shown. The corresponding 6AP derivatives were tested for their activity against the [*PSI* ^+^] prion (using the same strain and method as in Figure 1a and b). Note that only the 6AP derivative carrying the linker in position 3 (top panel) retains a significant antiprion activity. In the case of GA, the derivative carrying the linker in position 4 is still active. d. Molecular structures of 6AP (top) or GA (bottom) beads with the linker in position 3 on 6AP and 4 on GA, covalently linked to sepharose beads are shown.

6AP (6-aminophenanthridine, molecular structure depicted in **Figure 1a**) is a potent derivative of phenanthridine, a compound originally isolated from our laboratory chemical library as active against prions using the yeast-based assay described above [29], [30]. 6AP was found to be also active against mammalian prion in three mammalian cell-based assays ([29], [31] and **Figure 1a**, upper gel), namely the Rov system (rabbit kidney epithelial cells stably infected by the 127S sheep prion strain [32]), the N2a system (murine neuroblastoma cells stably infected by the RML murine prion strain [33], [34]) and the MovS6 system (immortalized neuroglial cells from ovine transgenic mice chronically infected by the 127S sheep prion strain [35]). In all these assays, PrP^Sc^ was detected on the basis of its proteinase K resistance. Guanabenz (GA, molecular structure depicted in **Figure 1b**) is an orally active central α2-adrenoceptor agonist already on the market for the treatment of hypertension [36]. Very recently, we identified this drug as active against both [*PSI* ^+^] and [URE3] yeast prions (Tribouillard *et al.*, in press and **Figure 1b**) as well as against mammalian prion, not only *ex vivo* in the MovS6-based assay described above but also *in vivo* in a mouse model previously described (tg338 mice infected with the 127S strain [37]).

In order to generate relevant controls, we reasoned that it could be of interest to design close derivatives of 6AP and GA lacking antiprion activity. During the course of structure-activity relationship (SAR) studies on the 6AP chemical family, numerous derivatives were synthesized. 6APi was obtained by introducing a 2-(butan-1-ol) group (depicted in blue in **Figure 1a**) on the 6-amino position of 6AP. 6APi is totally inactive against both [*PSI* ^+^] and [URE3] yeast prions and also against mammalian prion in the MovS6-based assays (**Figure 1a** lower panels). 6APi thus represents a proper negative control for experiments involving 6AP: despite its structural similarity with 6AP, it is totally inactive against both yeast and mammalian prions.

The SAR studies performed on the GA family suggested GAi is the most appropriate control molecule. GAi (**Figure 1b**, bottom structure) differs from GA by a single replacement of one of the two chlorine by a fluorine (also a halogen, highlighted in blue). This new molecule is totally inactive against yeast and mammalian prions (Tribouillard *et al.*, in press and **Figure 1b** lower panels).

### Design of 6AP and GA affinity matrices

In order to identify the cellular targets of 6AP and GA, we synthesized 6AP- and GA-linked affinity chromatography matrices to purify intracellular macromolecules able to physically interact with these drugs [38]. To avoid possible steric hindrance between target(s) and the matrix, we introduced a spacer between 6AP or GA and the sepharose beads. An aminocaproylaminopentyloxy linker was chosen because of its optimal length (F. G. and H. G., in preparation). In addition, its internal peptidic bond lowers its hydrophobicity, therefore reducing potential auto-aggregation or unspecific hydrophobic interactions with macromolecules from cell lysates. Furthermore, the amide function increases the rigidity of the linker. The position where this aminocaproylaminopentyloxy extension can be introduced while maintaining the antiprion activity of 6AP or GA was determined by testing the antiprion activity of their derivatives with the linker in various positions. Concerning 6AP, a previous SAR study has indeed shown that all but positions 1, 2 and 3 were crucial for its antiprion activity (F.G., N.D., M.B. and H.G., in preparation). Introduction of the aminocaproylaminopentyloxy linker in position 3 led to a reasonably active chemical derivative of 6AP, while the two other substitutions led to completely inactive compounds (**Figure 1c** upper panel). Similarly, introduction of the linker in position 4 of GA did not strongly affect its activity (**Figure 1c** lower panel). The branched 6AP and GA molecules were then bound to sepharose beads *via* the amino group of the linker to yield the affinity chromatography matrices depicted in **Figure 1d**. As control, sepharose beads quenched with ethanolamine were prepared. These matrices were then used in affinity chromatography experiments to identify cellular targets of 6AP or GA.

### 6AP and GA do not interact directly with prion proteins

Crude extracts of budding yeast, porcine brain or MovS6 (murine neuroglial) cells were prepared and incubated for 30 minutes with 6AP beads, GA beads or control beads. As additional controls, competitions with an excess of free 6AP or 6APi, respectively, or free GA or GAi, respectively, were performed, the rationale being that targets of the antiprion activity of 6AP or GA should be specifically competed away from the beads by 6AP or GA but not by 6APi or GAi, respectively. After extensive washing, bound proteins were eluted with SDS-PAGE sample buffer.

**Figure 2:**
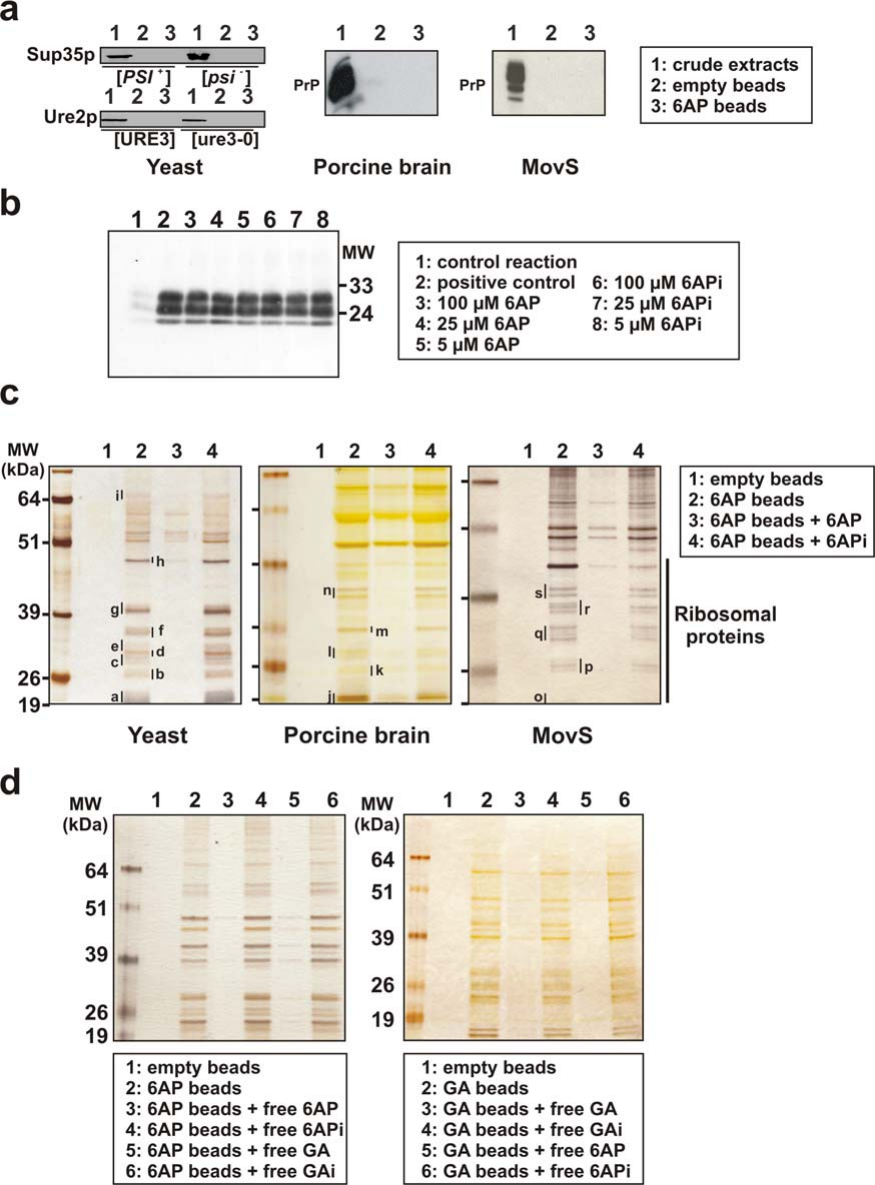
6AP and GA do not interact with prion proteins but show specific interactions with ribosomal components and compete with each other for interaction with ribosomal components. a. Extracts from yeast (left panel), porcine brain (middle panel) and murine MovS6 cells (right panel) were incubated with 6AP beads. The beads were then washed extensively and the bound proteins analyzed by SDS-PAGE followed by Western blotting analysis using antibodies directed against Sup35p, Ure2p or PrP as indicated. 1: crude extracts, 2: control beads without 6AP, 3: chromatography using 6AP beads. Note that none of the three prion proteins binds to 6AP beads. b. Protein Misfolding Cyclic Amplification (PMCA) reactions performed in the presence of various compounds and then subjected to proteinase K digestion followed by Western blot analysis using an anti-PrP antibody. All samples contain a mixture of normal and scrapie brain homogenates in addition to the tested compounds at various concentrations as indicated in the right panel. Note that both 6AP and 6APi are unable to inhibit *in vitro* conversion of PrP^C^ to PrP^Sc^. c. Extracts from yeast (left panel), porcine brain (middle panel) and murine MovS6 cells (right panel) were incubated with 6AP beads. The beads were then washed extensively and the bound proteins analyzed by SDS-PAGE. Lane 1: control beads without 6AP, lane 2: chromatography using 6AP beads, lane 3: competition with free 6AP, lane 4: competition with free 6APi, the inactive derivative of 6AP (see Figure 1a). Gels were silver-stained and specific bands were excised and analyzed by mass spectrometry. Note that for all the tested extracts, most of specific bands correspond to ribosomal proteins (see also table S1). d. Crude yeast cell extracts were incubated with 6AP beads (left gel) or GA beads (right gel). Lane 1: control beads without 6AP or GA, lane 2: chromatography using 6AP or GA beads, lanes 3 and 5: competition with free 6AP or GA, lanes 4 and 6: competition with free 6APi or GAi. The bound proteins were analyzed by SDS-PAGE followed by silver-staining. Note that both 6AP and GA (but not 6APi and GAi) are able to compete for the binding of ribosomal components to both 6AP and GA beads, suggesting that they share common binding site(s).

In principle, two mechanisms of action for antiprion drugs can be envisioned: either a *cis* mechanism, targeting prion protein/aggregates directly, or a *trans* mechanism, interfering with the activity of cellular factor(s) required for prion propagation. In the latter mechanism, antiprion drugs are not expected to interact directly with the prion proteins whereas the former mechanism implies direct interaction of the compound with the prion proteins either in their normal or prion form. Therefore proteins bound to 6AP beads were analyzed by SDS-PAGE followed by Western blotting using antibodies directed against Sup35p (the protein corresponding to [*PSI* ^+^] prion), Ure2p (the protein corresponding to [URE3] prion) or PrP. Although these three proteins were clearly detectable in crude cell extracts, they could not be detected among the components eluted from the 6AP beads after chromatography (**Figure 2a**), suggesting that there is no direct interaction between 6AP and these prion-forming proteins. This observation is consistent with the fact that 6AP is active against three prions ([*PSI* ^+^], [URE3] and PrP^Sc^) which do not share any significant sequence similarity. Moreover, no effect of 6AP or GA was observed on *in vitro* PrP^C^ to PrP^Sc^ conversion using a highly sensitive Protein Misfolding Cyclic Amplification (PMCA) method [39] (**Figure 2b** and data not shown), further strengthening the view that there is no direct interaction between prion proteins and 6AP or GA. Similarly, we found no significant effect of either 6AP and 6APi or GA and GAi on prion amyloid formation rate of purified recombinant Ure2p protein (**Figure S1a** and **c**). The same result was obtained using a second amyloid-forming prion protein, namely the prion forming domain (PFD) of the HET-s prion protein of *Podospora anserina* (**Figure S1b**), which is consistent with previous observations using an *in vitro* filter trap assay [40]. Taken together, these results suggest that 6AP and GA act *in trans* on cellular factors rather than *in cis* directly on the prion proteins.

### 6AP and GA show specific interactions with ribosomal components

Proteins bound to 6AP or GA beads were analyzed by SDS-PAGE followed by Coomassie blue (not shown) and silver staining (**Figure 2c**). Specific protein bands (absent from control beads, present on 6AP or GA beads and respectively competed away by free 6AP but not by free 6APi or by free GA but not by free GAi) were excised from the gel for identification by mass spectrometry. Most of the identified bands corresponded to ribosomal proteins from both the large and the small ribosomal subunits (**Table S1** and data not shown), suggesting that the ribosome or at least ribosome components could be specific targets of 6AP and GA. In the case of 6AP, some members of the Hsp70 family were also identified but only when using yeast extracts. These bound proteins (bands marked with an “i”) correspond to Ssa2p, Ssb1p and Ssb2p. Since these proteins were found to interact only with 6AP and not GA and only in yeast extracts and because they are described as ribosome-interacting proteins, interaction with these Hsp70 family members was not analyzed further.

We conclude that 6AP and GA, two chemically unrelated antiprion drugs isolated from two independent chemical libraries, appear to share the same cellular targets, namely components of the ribosome.

### 6AP and GA share common or overlapping binding sites on ribosomal components

We next tested whether 6AP and GA share the same interaction site(s) on ribosomal constituents by determining the ability of each compound to compete for the binding of ribosomal proteins to the other compound. As shown on lanes 5 and 6 of the left gel in **Figure 2d**, GA (but not GAi) is able to compete for the binding of ribosomal proteins to 6AP beads. Conversely, 6AP (but not 6APi) is able to compete for the binding of ribosomal proteins to GA beads (lanes 5 and 6, right panel). Taken together, these results suggest that 6AP and GA share common (or at least partially overlapping) binding site(s) on ribosomal components.

### 6AP and GA interaction with ribosomal components is RNA-dependent

**Figure 3:**
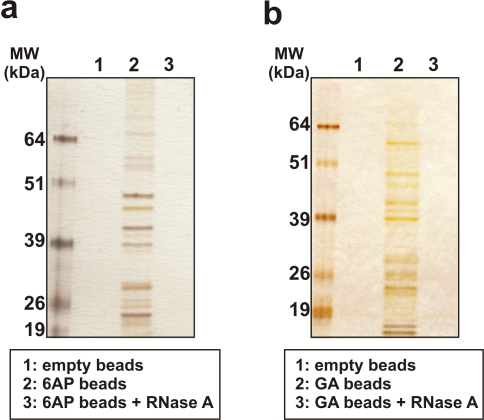
6AP and GA interaction with ribosomal components is RNA-dependent. Crude yeast cell extracts were incubated with 6AP beads (left) or GA beads (right). Lane 1: control beads without 6AP or GA, lane 2: chromatography using 6AP or GA beads, lane 3: cellular extracts were treated by RNase A at 100 µg/ml for 20 minutes at room temperature before affinity chromatography experiments. The bound proteins were analyzed by SDS-PAGE followed by silver staining. Note that after treatment of cellular extracts by RNase A, ribosomal constituents are unable to bind to 6AP or GA beads, indicating that interaction between both compounds and the ribosomal constituents is RNA-dependent.

Next we wondered whether the interaction of 6AP and GA with ribosomal proteins was dependent on RNA. We observed that an RNase A treatment of yeast crude extracts before affinity chromatography purification completely abolished the binding of the ribosomal proteins (and Hsp70s) to both 6AP (**Figure 3a**, lane 3) and GA beads (**Figure 3b**, lane 3). The same results were obtained using extracts from porcine brain or MovS6 cells (data not shown). We conclude that binding of ribosomal proteins to 6AP and GA is RNA-dependent. These results readily explain why a large number of distinct ribosomal proteins were retained by the affinity matrices.

### 6AP and GA have no effect on protein synthesis *in vivo* and *in vitro*

**Figure 4:**
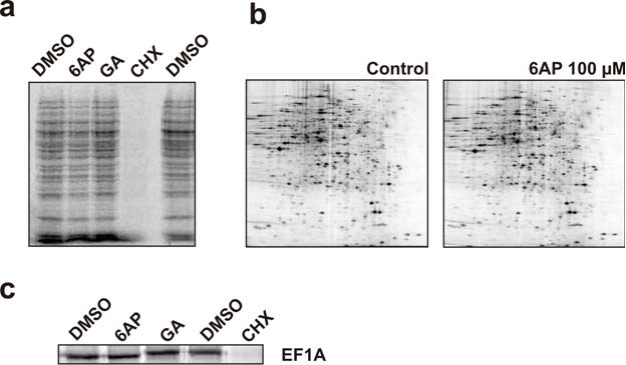
6AP and GA do not affect the translational activity of the ribosome both *in vivo* and *in vitro*. a. The effect of the indicated compounds on general *in vivo* translation in living yeast cells was evaluated. Briefly, various drugs or DMSO (the compounds vehicle) alone were added to yeast cells in exponential growth in YPD rich medium at a final concentration of 100 µM. After 10 minutes, radiolabelled [^35^S] methionine was added for 10 minutes. The cells were harvested to prepare cell extracts which were then analyzed by SDS-PAGE followed by autoradiography. Note that, with the exception of cycloheximide (CHX, a known inhibitor of general translation), none of the tested compounds has a significant effect on global protein synthesis. b. *WT* yeast cells were grown in the presence of DMSO (control left panel) or 100 µM 6AP (this concentration allows an efficient cure of prion) and cell extracts were then analyzed by 2D-SDS-PAGE followed by autoradiography. Note that no significant difference was observed between 6AP-treated cells and control cells. c. Effect of the indicated compounds on *in vitro* expression of EF1A using a commercial kit (Ambion). Note that only cycloheximide (CHX) significantly inhibits synthesis of EF1A.

Because the main function of the ribosome is protein synthesis, the effect of 6AP and GA on translation was assessed both *in vivo* and *in vitro*. Exponentially growing [*PSI* ^+^] yeast cells were treated with various compounds as indicated, at a final concentration of 100 µM (a concentration known to have an antiprion effect in yeast without any significant effect on the growth rate). Radiolabelled [^35^S] methionine was then added for 10 minutes and cells were harvested. Extracts were prepared and analyzed by SDS-PAGE (**Figure 4a**) or 2D-gel electrophoresis (**Figure 4b**) followed by autoradiography. None of the tested compounds exhibited a significant effect (inhibition or activation) on global protein synthesis. In addition, 6AP and GA had no significant specific effect on Sup35p, PrP or Ure2p protein level ([29] and data not shown). In sharp contrast, cycloheximide (CHX), a known inhibitor of global translation [41], completely inhibited protein synthesis at this concentration. The effect of 6AP and GA was also tested in an *in vitro* translation system based on rabbit reticulocyte lysate. The translation efficiency of a control mRNA encoding for the EF1A translation factor was evaluated in the presence of 200 µM of the indicated compounds (**Figure 4c**). Again, only CHX significantly inhibited EF1A mRNA translation. Therefore, at concentrations at which they exhibit antiprion activity, neither 6AP nor GA affects protein synthesis in these *in vivo* and *in vitro* systems.

### 6AP and GA inhibit the rRNA-mediated protein folding activity of the ribosome

**Figure 5:**
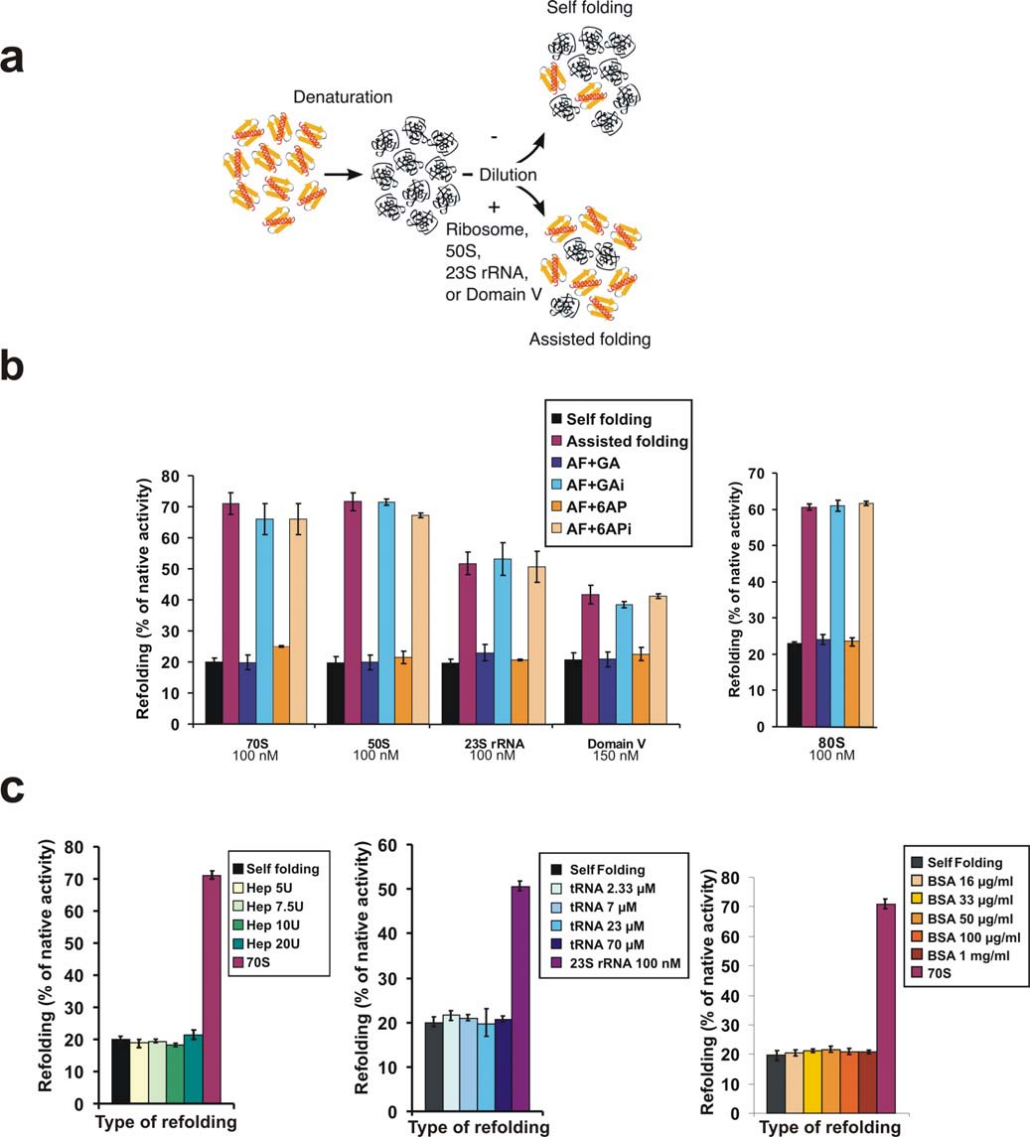
6AP and GA specifically inhibit the protein folding activity of the ribosome *in vitro*. a. Scheme depicting the principle of the *in vitro* assay to evaluate the protein folding activity of the ribosome. Human Carbonic Anhydrase (hCA) was denaturated (using GuHCl and EDTA) and then diluted into native buffer to allow refolding. Correct refolding was assessed by measuring the recovery of enzymatic activity as a function of time. Spontaneously, about 20% of the enzyme is able to refold correctly (Self folding, upper part of the scheme). If either a preparation of ribosomes, or the large subunit (50S), or the 23S rRNA, or the domain V of 23S rRNA, are added, the fraction of enzyme able to refold correctly increases up to 70%, due to the protein folding activity of the domain V of 23S rRNA of the large subunit of the ribosome (Assisted Folding -AF-, lower part of the scheme). b. Effect of the various drugs (as indicated) on assisted folding of hCA by eukaryotic (yeast *S. cerevisiae*, right panel) or prokaryotic (*E. coli* left panel) ribosomes (or indicated part of it). Concentration of each folding modulator is indicated. Note that only GA and 6AP, but not GAi and 6APi, were able to inhibit the assisted folding down to the level of self folding. c. Effect of heparin (left panel), tRNA (central panel) or BSA (right panel) on assisted folding of hCA. Note that none of these molecules is able to assist folding of hCA. Negative control is self folding and positive control is the assisted folding by 70S ribosome (for heparin and BSA experiments) or by 23S rRNA (for tRNA experiment).

In addition to its role in protein synthesis, the ribosome also assists protein folding. This ability has been demonstrated *in vitro* using bacterial as well as eukaryotic ribosomes and a variety of different proteins as substrates [15]–[17], [19]–[21], [23] and is supported by *in vivo* data in *E. coli* [18]. In all cases studied so far, this protein folding activity of the ribosome was shown to be mainly due to the domain V of the large rRNA (23S rRNA in *E. coli*) of its large subunit (50S in *E. coli*). This large rRNA, and particularly its domain V, is also involved in peptidyl transferase activity [42]. Since binding of 6AP and GA to the ribosome is RNA-dependent and exhibits no inhibitory effect on protein synthesis, we wondered if these drugs might modulate the protein folding activity of the ribosome. Human Carbonic Anhydrase (hCA) was used as a substrate for *in vitro* assisted folding experiments (**Figure 5**). The hCA protein was denaturated in the presence of guanidium hydrochloride and EDTA. For refolding experiments, hCA was diluted 100 times in native buffer either alone (to determine self-folding efficiency) or in the presence of highly active preparations of *E. coli* 70S ribosome, or the large subunit (50S), or rRNA purified from the 50S subunit (23S rRNA and 5S rRNA), or an *in vitro* transcribed 660 nucleotides long domain V from *B. subtilis* (see experimental scheme in **Figure 5a** and results in **Figure 5b**, left panel). The correct refolding of hCA was assessed by following the reappearance of its enzymatic activity. Self-folding restored about 20% of hCA activity. *E. coli* ribosomes or the 50S subunit alone restored about 70% of hCA activity, the 23S rRNA about 45% and the *in vitro* transcribed domain V about 35%. The extent of the assisted folding achieved by the different ribosomal folding modulators strongly depends on the structural integrity of the rRNA, which is probably better in 70S and 50S than in the phenol-extracted 23S rRNA and worse in the *in vitro* transcribed domain V. In control experiments, neither heparin (a control folding modulator with a negatively-charged backbone like RNA) nor tRNA (at concentration ranging from 2.33 µM to 70 µM which is several order of magnitude higher than the one at which ribosome or ribosomal components are efficient) were able to assist refolding of hCA, thus confirming that the protein folding activity of the large rRNA of the large subunit of the ribosome is specific and cannot be undertaken by other polyanions (**Figure 5c**, left and middle panels). The same result was obtained using BSA (right panel). 6AP and GA dramatically inhibited the ribosome-, 50S-, 23S- rRNA- and domain V-assisted folding to the level of self-folding (about 20%). This inhibition was specific since neither 6APi nor GAi showed any effect when added at the same concentration. Therefore, the potency of the four tested compounds (6AP, 6APi, GA and GAi) to inhibit the assisted folding activity of the ribosome parallels their activity as antiprion drugs. The same results were obtained using preparation of *S. cerevisiae* ribosome (**Figure 5b**, right panel) and other enzymes (porcine Malate Deshydrogenase and bovine Carbonic Anhydrase, data not shown) as substrates, suggesting that the ability of 6AP and GA to inhibit the protein folding activity of the ribosome is a general feature not restricted to bacterial systems.

We next determined whether the tested compounds could also affect the peptidyl transferase activity of the ribosome under the same experimental conditions (same preparation of *E. coli* ribosomes, same drugs and ribosome concentrations). As shown in **Figure S2a**, formation of the dipeptide (ML), a classical assay for peptidyl transferase activity [43], was not significantly affected by any of the four compounds. The same result was obtained in another experiment where the ability of ribosome to recycle was assessed, thus giving a global view on translation efficiency (data not shown).

**Figure 6:**
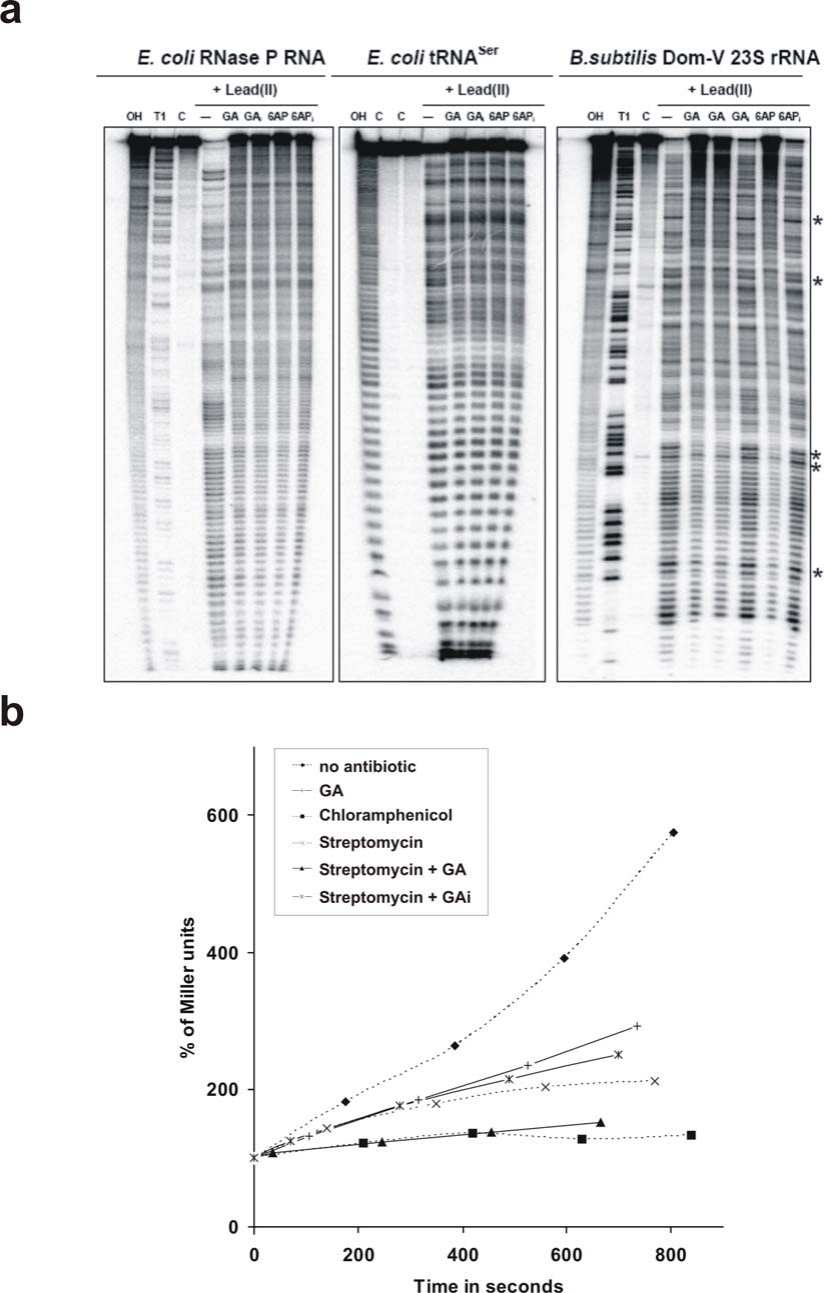
6AP and GA specifically bind to some common positions on domain V of large rRNA and inhibit the *in vivo* protein folding activity of the ribosome in *E. coli*. a. Chemical foot printing analysis of binding of 6AP and GA on various RNA. Asterisks indicate positions protected of degradation by Pb^2+^. Note that only 6AP and GA are able to protect some common positions specifically on Domain V of *B. subtilis* 23S rRNA (right gel) b. The increase in β-Gal activity was determined as a function of time in *E. coli* cells induced by IPTG and treated by the indicated compounds. Whereas both choramphenicol and streptomycin immediately and very efficiently inhibit translation, only chloramphenicol leads to an immediate inhibitory effect on increase in β-Gal activity, due to the fact that it also inhibits the protein folding activity of domain V of the large rRNA of the largest subunit of the ribosome. The increase observed in the presence of streptomycin corresponds to the folding by the ribosome of β-Gal translated just before translation inhibition by the antibiotic. This increase was inhibited by GA but not by GAi.

An *in vivo* assay of ribosomal protein folding activity has only been described in bacteria so far [18]. This assay is based on the comparison of the β-Galactosidase (β-Gal) activity in *E. coli* cells treated either by streptomycin or by chloramphenicol, both antibiotics inhibiting translation but having different effects on ribosomal protein folding activity *in vitro*. Streptomycin inhibits translation by binding to the small subunit of the bacterial ribosome and has thereby no effect on ribosomal folding activity *in vitro*, while chloramphenicol binds to the domain V of the 23S rRNA of the large subunit of the bacterial ribosome thus also inhibiting ribosomal protein folding activity *in vitro*. When *E. coli* cells are treated with either antibiotic, translation is totally inhibited as determined by pulse-chase assays ([18] and **Figure S2b**). In chloramphenicol-treated cells, the increase of β-Gal activity as a function of time immediately stops, while in streptomycin-treated cells, a significant (∼30%) increase of total β-Gal activity is observed. This difference was attributed to the fact that streptomycin-treated cells retain the protein folding activity of the ribosome and are thus able to properly fold the newly synthesized β-Gal molecules, i.e. synthesized just before the antibiotic totally inhibited translation ([18] and **Figure 6b**). The ability of 6AP, 6APi, GA or GAi to inhibit this 30% increase in β-Gal activity in streptomycin-treated *E. coli* cells was thus tested. Both 6AP and GA were able to efficiently inhibit the increase in β-Gal activity, whereas both 6APi and GAi were inactive (**Figure 6b** and data not shown), suggesting that 6AP and GA inhibited the *in vivo* protein folding activity of domain V. As controls, we checked that none of the four drugs inhibits translation, as judged by pulse-chase analysis (**Figure S2b**).

Taken together, these results demonstrate that 6AP and GA specifically inhibit the protein folding activity of the ribosome both *in vitro* and *in vivo* in *E. coli* without affecting its protein synthesis function.

### 6AP and GA specifically interact with common positions in domain V of the large rRNA of the large ribosomal subunit

In order to test the ability of 6AP and GA to directly interact with domain V of the large rRNA of the large subunit of the ribosome, we performed chemical foot-printing experiments on *in vitro* transcribed domain V of *B. subtilis* 23S rRNA as well as on two other completely unrelated *in vitro* transcribed RNAs, namely tRNA^Ser^ and RNAse P RNA. The results presented in **Figure 6a** show that none of the antiprion drugs (6AP and GA) nor their inactive analogues (6APi and GAi) were able to protect any region of tRNA^Ser^ and RNAse P RNA (left and middle panels). In contrast, foot-printing experiments on domain V of 23S rRNA showed specific and identical protection sites with both 6AP and GA but not with their inactive derivatives (right panel). These results clearly show a direct and specific binding of both 6AP and GA to the domain V of the 23S rRNA. Here again, the results obtained with the four compounds (6AP, 6APi, GA and GAi) parallel their respective activities as antiprion drugs.

## Discussion

In this article we show that the ribosome is a common intracellular target of 6AP and GA, two chemically unrelated antiprion drugs active from yeast to mammals. Importantly, these two drugs strongly and specifically inhibit the protein folding activity of the ribosome without affecting its protein synthesis activity. Ribosomal components were shown to interact specifically and in an RNA-dependent manner with both 6AP and GA. Control experiments using 6APi and GAi, two molecules structurally very close to 6AP and GA, but totally devoid of antiprion activity, highlight the specificity of 6AP and GA's interaction with the ribosome. Our results are best explained by postulating that in affinity chromatography experiments, the immobilized drugs retain the entire ribosome.

To our knowledge, 6AP and GA are the first reported compounds that selectively (i.e. without any significant effect on protein synthesis) inhibit the protein folding activity of the ribosome. Indeed, a number of antibiotics like chloramphenicol, lincomycin and erythromycin, which are known to bind the central large loop of domain V of 23S rRNA, inhibit the 23S rRNA/domain V-mediated protein folding activity but also strongly affect protein synthesis (thereby providing antibacterial activity) [20]. In contrast, antibiotics like streptomycin or kasugamycin, which bind to the 30S subunit of the ribosome and also block protein synthesis, have no effect on the protein folding activity of the ribosome which is borne by its 50S subunit. Therefore, as the first molecules specifically inhibiting the protein folding activity of the ribosome, 6AP and GA confirm that this activity is independent (or at least uncoupled) from its function in protein synthesis [25], [26]. In addition, these compounds constitute precious tools to study the yet largely unexplored biological role and significance of the protein folding activity of the ribosome *in vivo*.

At present, it cannot be excluded that, in addition to ribosome-assisted protein folding, 6AP and GA target other cellular processes that could account for their antiprion effect. However we detected no direct interaction between 6AP or GA and prion proteins. Moreover, all proteins identified in our affinity chromatography experiments are ribosome-associated. Importantly, we observe a strict correlation between the antiprion activity and inhibitory effect on ribosome-assisted folding of our different molecules. Therefore, although there is no direct evidence of a causal link between inhibition of ribosome-assisted folding activity and prion destabilization, the simplest interpretation of our results is that the domain V of the large rRNA is the functional target of both 6AP and GA. We thus propose that ribosome-assisted protein folding might be involved in prion propagation both in yeast and mammals. Since ribosome structure and function are largely conserved throughout evolution, this hypothesis would account the universality of the antiprion effect of 6AP and GA. Of note is the fact that RNA molecules strongly increase PrP transconformation *in vitro* [44], [45]. Indeed, total hamster brain RNA fractionation experiments showed that RNA molecules of more than 300 nucleotides (that co-purify with rRNA) retain this amplification ability whereas smaller RNA do not. In addition, the putative function of the largest rRNA in prion formation/propagation provides mechanistic support for previously unexplained observations in yeast showing the ability of 3 µm plasmid (encoding rRNA) to induce [*psi^−^*] to [*PSI* ^+^] conversion when introduced into a [*psi* ^−^] strain [46]. Very recently, it has been shown that in *Drosophila melanogaster*, overexpression of chaperone-like highly structured RNA induces congophilic aggregates and facilitates neurodegeneration [47], also suggesting that a highly structured RNA alone could be able to trigger neurodegeneration through chaperone-like facilitation of protein misfolding and aggregation.

If the causal link between down-regulation of protein folding activity of the ribosome and prion destabilization remains to be established, as the first molecules that specifically affect protein folding activity of the ribosome, 6AP and GA already stand out as prime new tools to decipher the *bona fide* biological role(s) of this chaperone-like activity of the ribosome.

## Materials and Methods

### Yeast strains, yeast-based antiprion assay and genetic manipulations

Yeast strains used in this study were as follows. 74-D694: *Mata*, *ade1-14*, *trp1-289*, *his3*Δ*200*, *ura3-52*, *leu2-3,112*, [*PSI* ^+^] [27], Strg6: *Mata*, *erg6::TRP1*, *ade1-14*, *trp1-289*, *his3*Δ*200*, *ura3-52*, *leu2-3,112*, [*PSI* ^+^] and SB34: *Mata*, *erg6::TRP1*, *dal5::ADE2*, *ade2-1*, *trp1-1*, *leu2-3,112*, *his3-11,15*, *ura2::HIS*, [URE3] [29] and were grown and used as described [30].

### Yeast-based antiprion screening assay

This assay was done as previously described [29], [30]. Briefly, yeast cells containing either [*PSI*+] or [URE3] prion lead to the formation of white colonies on rich (YPD) medium whereas, once cured of these prions ([*psi*−] or [ure3-0] cells), they lead to the formation of red colonies, due to the accumulation of a metabolic byproduct of the adenine biosynthesis pathway in *ade1-* or *ade2-* cells. In the case of the [*PSI*+] prion, this accumulation is decreased in [*PSI*+] cells due to the increased tendency of ribosome to read through stop codon of the *ade1-14* mutant allele of the *ADE1* gene, caused by sequestration of most of Sup35p (a termination factor homolog of eRF3 in mammals) in prion aggregates. In the case of the [URE3] prion, the accumulation of the metabolic byproduct is decreased in [URE3], *ade2-* cells due to the expression of a *WT* copy of the *ADE2* gene under the control of the *DAL5* gene promoter which is normally repressed in [ure3-0] cells [29], [48]. An aliquot (350 µl of an 0.5 OD_600_ overnight culture) of [*PSI*+] or [URE3] cells (which grow as white colonies) were spread using sterile glass beads on square (12 cm×12 cm) Petri plates containing YPD medium supplemented with 200 µM Guanidine hydrochloride (-GuHCl- conditions where the sensitivity of the method is optimal). Sterile small filters (similar to the ones used for antibiograms) were then placed on the agar surface and individual compounds were applied to each filter. The Petri plates were then incubated three days at 25°C. When a compound is active against [*PSI*+] or [URE3] prions, a halo of red colonies appear around the filter where it was spotted whereas colonies remain white in case of inactive compounds. To confirm that potentially active compounds really cure yeast prions and do not act against the colorimetric system used as a reporter, cells from the red halos were streaked on a fresh drug-free YPD medium to control that they still form red colonies, an indication that [*PSI*+] or [URE3] prions were actually cured in these cells [30], [31].

### PrP^Sc^ inhibition assay in MovS6 cells

Scrapie (127S strain)-infected neuroglial cells (MovS6 [35]) were treated for 6 days at 37°C, 5% CO_2_ with 0 to 20 µM of the indicated drugs or, as controls by the corresponding volume of DMSO. Cells extracts were then digested with proteinase K and analyzed by Western blotting using antibody Sha31 (mouse, dilution 1/5000) directed against PrP protein [49]. Quantification of remaining PrP^Sc^ compared to controls was done using the Vilber-Lourmat Photodocumentation Chemistart 5000 imager.

### Preparation of 6AP- and GA- resins

The preparation of the conjugates of 6AP and GA, depicted respectively in **Figures 2** and **3** will be published elsewhere (Gug *et al.*, submitted). The conjugates were coupled in 0.2 M NaHCO_3_, 0.2 M NaCl, pH 8.5 on Fast flow CNBr Activated Sepharose 4B® supplied by Amersham. The final calculated concentration was 5–25 µmol/ml of resin. The remaining active group were blocked in pH 8.0 buffer containing 1 M ethanolamine.

### Preparation of cell extracts and affinity chromatography on immobilized drugs

The homogenization buffer for yeast cell extracts was 25 mM Tris (pH 7.4), 100 mM NaCl, 0.2% Triton X100 and 1 mM PMSF. The homogenization buffer for porcine brain extracts was 60 mM β-glycerophosphate, 15 mM *p*-nitrophenyl phosphate, 25 mM MOPS (pH 7.2), 15 mM EGTA, 15 mM MgCl2, 1 mM sodium vanadate, 1 mM NaF, 1 mM dithiothreitol, 1 mM phenyl phosphate, 10 µg/ml leupeptin, 10 µg/ml aprotinin, 10 µg/ml of soybean trypsin inhibitor and 100 µM benzamidine. The homogenization buffer for Mov cell extracts was 50 mM Tris (pH 7.4), 0.5% sodium deoxycholate, 0.5% Triton X100. At the end of the preparation procedure, yeast RNA extracts were resuspended in water treated by Di Ethyl Pyro Carbonate (DEPC).

Yeast protein extracts were obtained from a culture growing at 30°C (OD_600 nm_ = 0.8). Cell pellets were resuspended in homogenization buffer (300 µl/100 ml of culture) and lysed using acid washed glass beads (purchased from Sigma, 300 µl/100 ml of culture). Homogenates were vortexed for 30 sec followed by 30 sec ice-cooling (six times) and then centrifuged for 3 min at 3,000 rpm at 4°C. Supernatants were recovered, assayed for protein content (using Bio-Rad protein assay) and immediately loaded batchwise on the affinity matrix.

Porcine brain extracts were obtained from a local slaughterhouse (Louis Gad SA) and directly homogenized and processed for affinity chromatography or stored at −80°C prior to use. Tissues were weighed, homogenized and sonicated in homogenization buffer (2 ml/g of material). Homogenates were centrifuged for 10 min at 14,000× *g* at 4°C. The supernatants were recovered, assayed for protein content (using Bio-Rad protein assay) and immediately loaded batchwise on the affinity matrix.

MovS6 cell extracts were obtained from a culture grown at 37°C, 5% CO_2_, washed with PBS 1× and homogenized in homogenization buffer (1 ml for a 25 cm^2^ culture flask). Homogenates were incubated for 10 min at 4°C and centrifuged for 1 min at 2,000 rpm at 4°C. The supernatants were recovered, assayed for protein content (Micro BCA, Pierce) and immediately loaded batchwise on the affinity matrix.

Just before use, 6AP and GA beads were washed with 1 ml of bead buffer (50 mM Tris (pH 7.4), 5 mM NaF, 250 mM NaCl, 5 mM EDTA, 5 mM EGTA, 0.1% Nonidet P-40, 10 µg/ml of leupeptin, aprotinin, and soybean trypsin inhibitor, and 100 µM benzamidine) and diluted 4 times in this buffer. Protein extracts: 200 µg of yeast protein extract, or 2 mg of porcine brain or MovS6 cell protein extract were added to 40 µl of diluted beads (10 µl of packed beads). The volume was adjusted to 600 µl by adding bead buffer, the tubes were rotated at 4°C for 30 min. Competitions with free active or inactive drugs were performed with free compounds (or the corresponding volume of DMSO) at a final concentration of 1 mM incubated with cell extracts for 3 min before the mixture was added to the affinity matrix. Then, bead buffer containing the same quantity (1 mM) of free drugs was immediately added (as described previously) to reach a final volume of 600 µl. After a brief spin at 10,000× *g* at 4°C and removal of the supernatant, the beads were washed 4 times with bead buffer before addition of 35 µl of 2× Laemmli sample buffer. Following heat denaturation for 3 min at 95°C, the bound proteins were analyzed by SDS-PAGE and Western blotting or silver staining as described below.

### Electrophoresis, Silver staining, Western blots and antibodies

Following heat denaturation for 3 min at 95°C, the proteins bound to 6AP or GA matrices were separated by 10% SDS-PAGE (precast NuPAGE -Invitrogen- 1 mm thick gel) followed by immunoblotting analysis or silver staining using an Amersham SDS-PAGE silver staining kit. For immunoblotting, proteins were transferred to 0.45 µm nitrocellulose filters (Schleicher and Schuell). These were blocked with 5% skimmed milk in Tris-buffered saline/Tween 20, incubated for 1 H with the indicated antibodies, and analyzed by Enhanced Chemiluminescence (ECL, Amersham) using a Vilber-Lourmat Photodocumentation Chemistart 5000 imager.

Antibody anti-PrP used: Sha31 (mouse, dilution 1/5000) [49]. Antibody anti-Sup35p used: rabbit polyclonal raised against peptide 55–68 of Sup35p, a kind gift of S. L. Lindquist, dilution 1/1000. Antibody anti-Ure2p used: rabbit polyclonal anti-Ure2p, a kind gift of L. Maillet, dilution 1/5000.

### PMCA assays

All tested compounds were resuspended in DMSO to make 12.5 mM stock solutions and stored at −20°C prior to use. Working solutions were prepared by serially diluting the stock solution into water.

To make normal mouse brain homogenate, two frozen mouse brains (Harlan Sprague Dawley, Inc., Indianapolis, IN) were Potter homogenized in 10 ml of ice-cold PBS (phosphate-buffered saline without calcium or magnesium) containing Complete protease inhibitors (Roche, Indianapolis, IN). The homogenate was centrifuged at 200× *g* for 30 sec, and the post-nuclear supernatant was collected.

Protein Misfolding Cyclic Amplification (PMCA) reactions consisted of 50 µl 10% normal mouse brain homogenate, 40 µl RML scrapie brain homogenate (diluted 1/200 into PBS, 1% Triton X-100, 5 mM EDTA), and 10 µl tested compounds or control buffer (8% DMSO in water). Intermittent sonication was performed in 0.5 ml thin-walled PCR tubes using a Misonix 3000MPD device, using a variation of the semi-automated procedure originally described [50]. The sonicator output setting was 6.0, and a 30 sec pulse was delivered every 30 min for 24 H. The sonicator horn was filled with 350 ml water, which was maintained at 41°C.

Following PMCA reactions, all samples were treated with 25 µg/ml proteinase K for 1 H at 37°C, subjected to SDS-PAGE, and transferred onto a PVDF membrane which were subsequently probed with monoclonal antibody 6D11 (Signet, Dedham, MA) at 1/15000 dilution in TBST.

### MALDI-TOF Peptide Mapping Protein Identification

The protein bands were excised from a one-dimensional SDS-PAGE gel stained by Coomassie blue and digested in gel with trypsin as described previously [51]. MALDI analysis and interpretation were realized by Innova Proteomics (Rennes, France).

### Translation assays

The dipeptide formation assay was designed following [43], in a cell-free bacterial translation system made up of highly purified components from *E. coli* [52]. An initiation complex (IC) containing *E. coli* 70S ribosome, [^3^H]f-Met tRNA^f-Met^, an mRNA coding for fMet-Leu-Ile-stop, and all three bacterial initiation factors was prepared, with or without the indicated drugs (6AP, 6APi, GA or GAi) at a final concentration of 1 mM. In parallel, a ternary complex (TC) containing Leucine, tRNA^Leu^, Leu-tRNA synthetase, EF-Tu, EF-Ts and GTP was prepared. The reaction was initiated by rapid mixing of the IC and TC in a quench-flow instrument. The reaction was quenched at different time-points by addition of formic acid, then the amount of [^3^H]f-Met-Leu dipeptide was quantified in HPLC equipped with an on-line radio detector [53]. *In vitro* translation assay based on the rabbit reticulocyte lysate (Ambion kit) was used according to the instruction of the manufacturer. Translation of the control capped mRNA (encoding for EF1α from *Xenopus laevis*) provided in the kit was evaluated in presence of the various compounds all at a final concentration of 200 µM. Autoradiography and quantification were made using a Storm 840 Phosphorimager (Molecular Dynamics).

A first *in vivo* translation assay was performed using a yeast culture grown at 30°C in YPD (OD_600 mm_ = 0.6 in exponential phase of growth) incubated with the indicated molecules (100 µM) or the corresponding volume of DMSO (compound vehicle) for 10 min at 30°C at which time [^35^S] methionine was added for 10 min. Crude yeast extracts were then realized as described previously and analyzed by SDS-PAGE. The gel was dried and analyzed using a Storm 840 Phosphorimager (Molecular Dynamics). A second *in vivo* translation assay was performed as previously described [54] and analyzed by two dimensional SDS-PAGE.

### *In vitro* ribosome assisted folding assays

*E. coli* ribosome, *S. cerevisiae* ribosome and 50S subunit were prepared using sucrose gradient zonal ultracentrifugation as described previously [55]. 23S rRNA was isolated from 50S subunit by 8 successive phenol-chloroform (1:1) extractions. The aqueous phase containing rRNA was precipitated by addition of 2 volumes ethanol and 0.1 volume of 5 M sodium acetate pH 5.2. As the 23S rRNA from different bacterial species has identical secondary structure, especially in the central loop of the domain V, we have chosen to transcribe *in vitro* the 660 nt long domain V of 23S rRNA, due to the availability of the respective clone in plasmid pDK105 [56]. The plasmid was linearised using *Eco*RI and the run off RNA was then transcribed from the SP6 promoter using SP6 RNA polymerase (GE Healthcare). The DNA template was digested with RNase-free Dnase I and RNA was precipitated with ethanol after phenol-chloroform extraction. Further the RNA concentration was evaluated using Nanodrop spectrophotometer. The bulk tRNA was purified as described [57]. Heparin was purchased from Lovens Kemiske Fabrik (Denmark) and BSA from Promega.

For the *in vitro* refolding experiments, human Carbonic Anhydrase (hCA) at a concentration of 20 µM was denaturated using guanidium hydrochloride 6 M and EDTA 30 nM. To allow refolding, hCA was then diluted 100 times (final concentration 200 nM) in a buffer containing 20 mM Tris HCl (pH 7.5), 100 mM NaCl and 5 mM magnesium acetate for 30 min with or without the folding modulators (each at 100 nM final concentration, except for the *in vitro* transcribed domain V which is at 150 nM) and/or the drugs (all at 1 mM final concentration). The refolding of hCA, as a function of its activity, was followed by the colorimetric assay measuring the increase of A_400_ with time when the substrate of hCA, para-nitrophenyl acetate (pNPA), was added at a final concentration of 500 µM directly to the refolding mix.

### *In vivo* ribosome assisted folding assays

E. coli strain (MRE600 [18]) was grown in LB medium to an OD_600 nm_ of 0.15 at which time β-Gal expression was induced by adding IPTG to a final concentration of 1 mM. The culture was then splitted in different flasks in which 6AP, or 6APi (both at 100 µM final concentration), GA or GAi (both at 200 µM final concentration) were added. After one hour of incubation, streptomycin (17 µM final concentration), or chloramphenicol (464 µM final concentration) or the corresponding volume of DMSO were added to the indicated cultures. At the indicated times, 1 ml of the various cultures was collected. Bacteria were lyzed by mixing immediately 500 µl of collected fractions to 500 µl of toluene whereas the remaining 500 µl were used to determine OD_600 nm_. Bacteria in toluene were vortexed for 30 sec. and then left at room temperature before an additional round of vortexing. The aqueous phases were then collected and the levels of β-Gal activity were determined as previously described [58]. Miller units were calculated using the formula [(1000* OD_420 nm_−(1.75* OD_550 nm_))]/[time of reaction in min.*volume of lysate in ml* OD_600 nm_].

### Chemical foot printing analysis of binding of 6AP and GA to various RNA

The chemical footprinting analysis were performed in the presence of freshly prepared PB(OAc)_2_ solution as previously described [59]. *E. coli* RNase P RNA and tRNA^Ser^, and domain V of *B. subtilis* 23S rRNA were transcribed *in vitro* and labeled with ^32^P-pCp at the 3′ end. The RNAs were subjected to Pb^2+^-hydrolysis (5 mM) for 5 to 7 mins, in the absence or presence of the antiprion drugs 6AP and GA or their inactive derivatives 6APi and GAi respectively, at a final concentration of 1 mM in DMSO. The alkaline hydrolysis and the G-specific RNase T1 digestion were performed under denaturating conditions, according to the manufacturer's protocol (Ambion). The footprint as well as the control assays (DMSO-treated) were separated on denaturing 8% polyacrylamide gels and further analysed using a Phosphorimager (Molecular Dynamics 400S).

## Supporting Information

Table S1 (0.02 MB PDF)

Figure S1 Amyloid aggregation of purified fungal prion proteins in the presence of 6AP and GA and their inactive derivatives. We have analyzed the amyloid formation rate of purified recombinant Ure2p yeast prion protein in the presence of 6AP and GA and their inactive derivatives (Figure S1a). Aggregation kinetics were followed by light scaterring. Fibril formation was verified at polymerisation end points by ThT fluoresence (not shown) and Ure2p fibril morphology was also analyzed by EM. There is currently no molecule described to inhibit Ure2p amyloidogenesis that could have been used as a positive control in these experiments but since Congo red was decribed as an antiprion drug acting in cis and is known to delay amyloid formation in some systems [1], we chose to also included it in this experiment. Neither 6AP nor GA affected prion amyloid formation rate of Ure2p significantly while CR had a slight inhibitory effect. 6APi also induced a modest delay in amyloid formation. However, the compound was not fully soluble in the used conditions. Ure2p fibril morphology was the same with either 6AP, GA or their inactive derivatives (Figure S1c). The same experiment was also performed with the prion forming domain of HET-s, a fungal prion protein. There again, neither 6AP nor GA affected significantly amyloid formation. Once more, the only compound that exerted a significant effect were CR and 6APi which here accelerated aggregation significantly (Figure S1b). It has been described previously that CR can have an inhibitory or pro-aggregative effect depending on the considered peptide or protein [1]. Amyloid formation rate of the Ure2p and HET-s PFD were monitored at pH 7 and 37°C in the presence of antiprion drugs and inactive derivatives. Prion aggregation was monitored by measuring the scattering at 600 nm. The kinetics of the aggregation at 10 µM of protein in absence or in the presence of 1 mM of GA, GAi, 6AP, 6APi or 0.01 mM of Congo Red (CR) were determined and the half-aggregation times have been obtained for (a) Ure2p aggregation, (b) HET-s PFD aggregation. (c) Ure2p amyloids have been analyzed at reaction end points by electron microscopy, scale bar is 100 nm. In these experiments, we found no correlation between the antiprion activity of the GA and 6AP and their inactive derivatives and their effect in cis on prion amyloid formation using purified recombinant proteins. 1. Frid P, Anisimov SV, Popovic N (2007) Congo red and protein aggregation in neurodegenerative diseases. Brain Res Brain Res Rev 53: 135–160.
(2.68 MB TIF)

Figure S2 6AP and GA antiprion drugs do not inhibit protein synthesis. a - Effect of antiprion drugs on in vitro translation. The formation of f-Met-Leu dipeptide was assayed in an in vitro translation system based on purified E. coli ribosome in the presence of 1 mM of 6AP, 6APi, GA and GAi. None of the tested drugs showed a significant effect on the kinetics of translation. b - Effect of antiprion drugs on general in vivo translation in living E. coli cells. E. coli strain (MRE600 [1]) was grown in LB medium to an OD600 nm of 0.15 at which time β-Gal expression was induced by IPTG. Bacteria were then incubated in the presence of 100 µM of 6AP or 6APi, 200 µM of GA or GAi, streptomycin (17 µM) or chloramphenicol (464 µM) as described in the material and methods section (paragraph “In vivo ribosome assisted folding assays”). Cells were then incubated in the presence of radiolabelled [35S] methionine for 10 minutes, harvested and lysed in RIPA buffer. Equivalent quantities of cell lysates were analyzed by SDS-PAGE followed by autoradiography. 1. Chattopadhyay S, Pal S, Pal D, Sarkar D, Chandra S, et al. (1999) Protein folding in Escherichia coli: role of 23S ribosomal RNA. Biochim Biophys Acta 1429: 293–298.
(0.86 MB TIF)
