# Supplementary material for: Protein Folding Activity of Ribosomal RNA Is a Selective Target of Two Unrelated Antiprion Drugs
Source: PLoS One. 2008 May 14;3(5):e2174. doi: 10.1371/journal.pone.0002174 (PMC2374897; doi:10.1371/journal.pone.0002174)
Supplement: Table S1 — (0.02 MB PDF) [file pone.0002174.s001.pdf]

Table S1

Mass spectrometry identification of proteins bound to 6AP beads

Group number	Origin	Theoretical molecular weight (kDa)
	**Yeast**	
a.	*RPS11, RPL20, RPL24B, RPL24A*	18, 21, 18, 18
b.	*RPS9A, RPS9B, RPL18, RPL6A, RPL6B*	23, 23, 21, 20, 20
c.	*RPL19, RPS7A, RPL16A*	23, 23, 23
d.	*RPS8, RPL10, RPL7A, RPL7B, RPL15A*	25, 26, 28, 28, 25
e.	*RPS4,RPS1A, RPS1B, RPL8A, RPL2*	29, 29, 29, 28, 28
f.	*RPL5, RPLA0*	34, 34
g.	*RPL4A, RPL4B*	40, 40
h.	*RPL3*	45
i.	*SSA2, SSB1, SSB2*	69, 67, 67
	**Porcine brain**	
j.	*RPL7A*	16
k.	*RPL18, RPS5*	22, 22
l.	*RPL10, RPL13, RPL14*	25, 25, 25
m.	*RPL5*	35
n.	*RPL3*	47
	**MovS**	
o.	*RPL23*	18
p.	*RPL18, RPL13, RPL14*	22, 25, 24
q.	*RPS2, RPL7A*	29, 31
r.	*RPL6, RPP0, RPS8*	33, 34, 37
s.	*RPL3*	48
