# Supplementary figures and images for: Protein Folding Activity of Ribosomal RNA Is a Selective Target of Two Unrelated Antiprion Drugs
Source: PLoS One. 2008 May 14;3(5):e2174. doi: 10.1371/journal.pone.0002174 (PMC2374897; doi:10.1371/journal.pone.0002174)

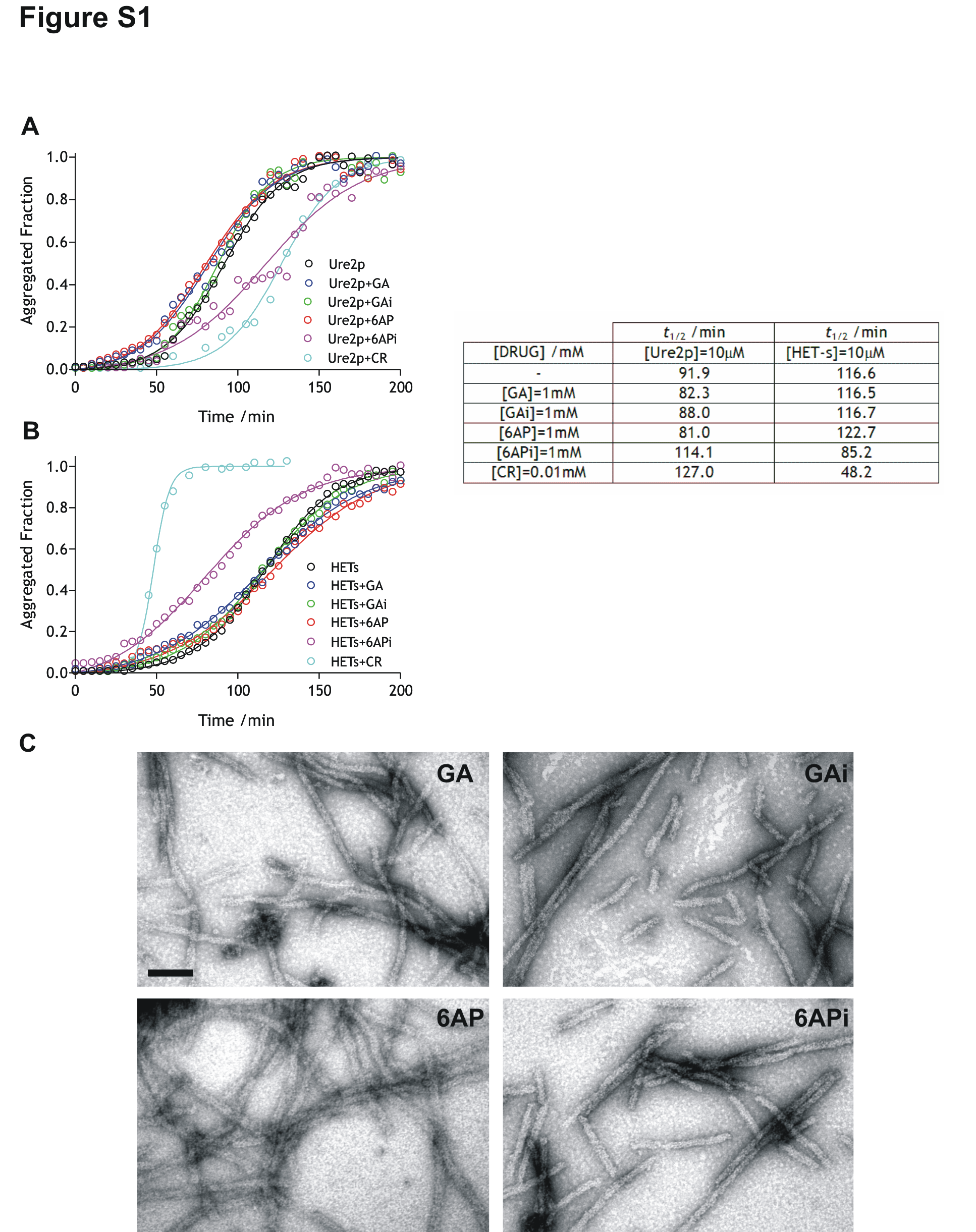

Supplement: Figure S1 — Amyloid aggregation of purified fungal prion proteins in the presence of 6AP and GA and their inactive derivatives. We have analyzed the amyloid formation rate of purified recombinant Ure2p yeast prion protein in the presence of 6AP and GA and their inactive derivatives (Figure S1a). Aggregation kinetics were followed by light scaterring. Fibril formation was verified at polymerisation end points by ThT fluoresence (not shown) and Ure2p fibril morphology was also analyzed by EM. There is currently no molecule described to inhibit Ure2p amyloidogenesis that could have been used as a positive control in these experiments but since Congo red was decribed as an antiprion drug acting in cis and is known to delay amyloid formation in some systems [1], we chose to also included it in this experiment. Neither 6AP nor GA affected prion amyloid formation rate of Ure2p significantly while CR had a slight inhibitory effect. 6APi also induced a modest delay in amyloid formation. However, the compound was not fully soluble in the used conditions. Ure2p fibril morphology was the same with either 6AP, GA or their inactive derivatives (Figure S1c). The same experiment was also performed with the prion forming domain of HET-s, a fungal prion protein. There again, neither 6AP nor GA affected significantly amyloid formation. Once more, the only compound that exerted a significant effect were CR and 6APi which here accelerated aggregation significantly (Figure S1b). It has been described previously that CR can have an inhibitory or pro-aggregative effect depending on the considered peptide or protein [1]. Amyloid formation rate of the Ure2p and HET-s PFD were monitored at pH 7 and 37°C in the presence of antiprion drugs and inactive derivatives. Prion aggregation was monitored by measuring the scattering at 600 nm. The kinetics of the aggregation at 10 µM of protein in absence or in the presence of 1 mM of GA, GAi, 6AP, 6APi or 0.01 mM of Congo Red (CR) were determined and the half-aggr [file pone.0002174.s002.tif]

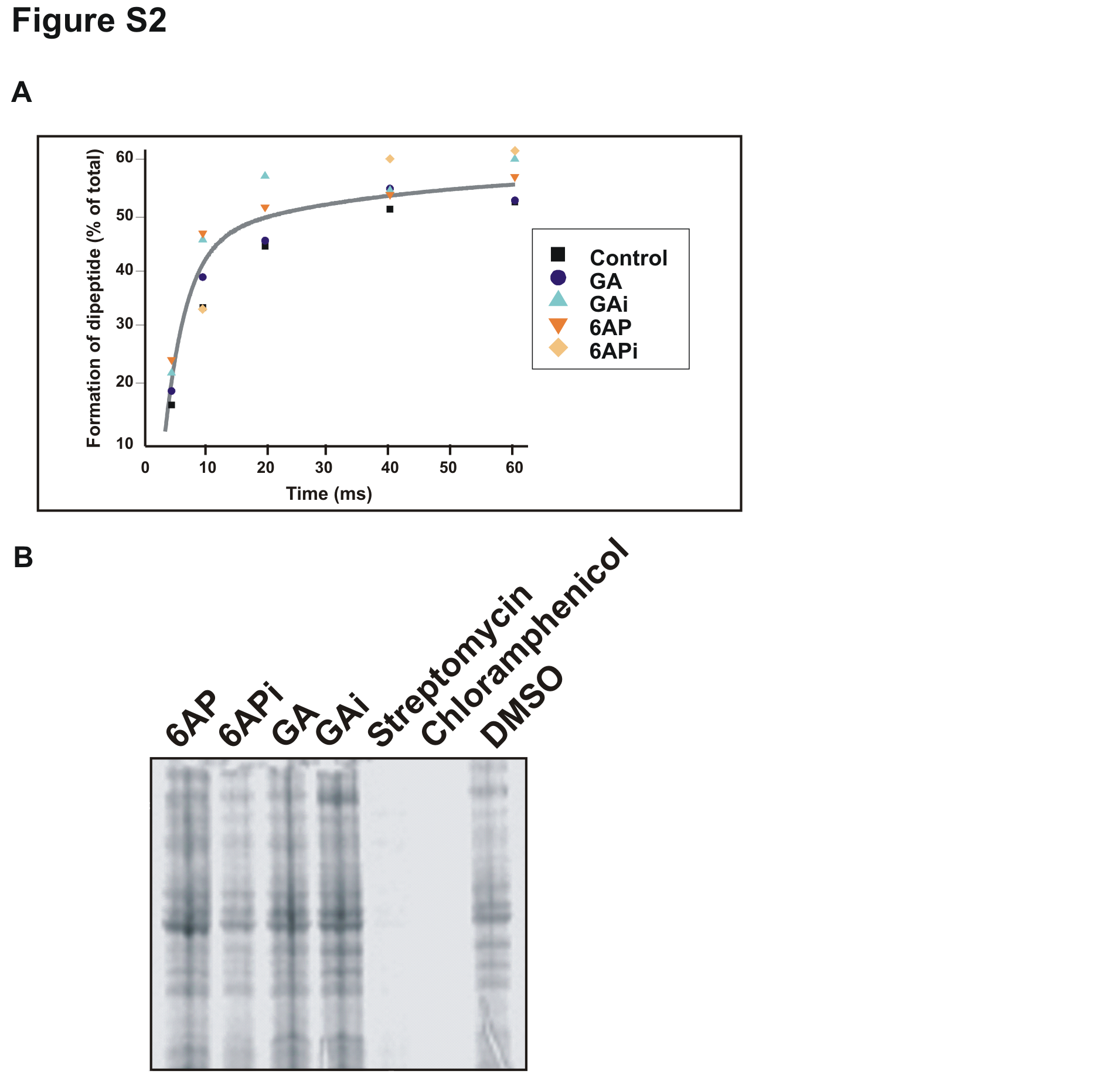

Supplement: Figure S2 — 6AP and GA antiprion drugs do not inhibit protein synthesis. a - Effect of antiprion drugs on in vitro translation. The formation of f-Met-Leu dipeptide was assayed in an in vitro translation system based on purified E. coli ribosome in the presence of 1 mM of 6AP, 6APi, GA and GAi. None of the tested drugs showed a significant effect on the kinetics of translation. b - Effect of antiprion drugs on general in vivo translation in living E. coli cells. E. coli strain (MRE600 [1]) was grown in LB medium to an OD600 nm of 0.15 at which time β-Gal expression was induced by IPTG. Bacteria were then incubated in the presence of 100 µM of 6AP or 6APi, 200 µM of GA or GAi, streptomycin (17 µM) or chloramphenicol (464 µM) as described in the material and methods section (paragraph “In vivo ribosome assisted folding assays”). Cells were then incubated in the presence of radiolabelled [35S] methionine for 10 minutes, harvested and lysed in RIPA buffer. Equivalent quantities of cell lysates were analyzed by SDS-PAGE followed by autoradiography. 1. Chattopadhyay S, Pal S, Pal D, Sarkar D, Chandra S, et al. (1999) Protein folding in Escherichia coli: role of 23S ribosomal RNA. Biochim Biophys Acta 1429: 293–298. (0.86 MB TIF) [file pone.0002174.s003.tif]
